# Oral Administration of Valganciclovir Reduces Clinical Signs, Virus Shedding and Cell-Associated Viremia in Ponies Experimentally Infected with the Equid Herpesvirus-1 C_2254_ Variant

**DOI:** 10.3390/pathogens11050539

**Published:** 2022-05-04

**Authors:** Côme J. Thieulent, Gabrielle Sutton, Marie-Pierre Toquet, Samuel Fremaux, Erika Hue, Christine Fortier, Alexis Pléau, Alain Deslis, Stéphane Abrioux, Edouard Guitton, Stéphane Pronost, Romain Paillot

**Affiliations:** 1 LABÉO, 14280 Saint-Contest, France; cthieulent@lsu.edu (C.J.T.); gabrielle.sutton@laboratoire-labeo.fr (G.S.); marie-pierre.toquet@laboratoire-labeo.fr (M.-P.T.); samuel.fremaux@laboratoire-labeo.fr (S.F.); erika.hue@laboratoire-labeo.fr (E.H.); christine.fortier@laboratoire-labeo.fr (C.F.); stephane.pronost@laboratoire-labeo.fr (S.P.); 2 BIOTARGEN EA 7450, Normandie University, UNICAEN, 14280 Saint-Contest, France; 3 Louisiana Animal Disease Diagnostic Laboratory and Department of Pathobiological Sciences, School of Veterinary Medicine, Louisiana State University, Baton Rouge, LA 70803, USA; 4 ImpedanCELL, Normandie University, UNICAEN, 14280 Saint-Contest, France; 5 INRAE, UE-1277 Plateforme D’infectiologie Expérimentale (PFIE), Centre de Recherche Val de Loire, 37380 Nouzilly, France; alexis.pleau@inrae.fr (A.P.); alain.deslis@inrae.fr (A.D.); stephane.abrioux@inrae.fr (S.A.); edouard.guitton@inrae.fr (E.G.); 6 School of Equine and Veterinary Physiotherapy, Writtle University College, Lordship Road, Writtle, Chelmsford CM1 3RR, UK

**Keywords:** valganciclovir, ganciclovir, EHV-1, equid alphaherpesvirus-1, herpesvirus, antiviral, experimental infection, pony

## Abstract

Equid alphaherpesvirus-1 (EHV-1) is one of the main pathogens in horses, responsible for respiratory diseases, ocular diseases, abortions, neonatal foal death and neurological complications such as equine herpesvirus myeloencephalopathy (EHM). Current vaccines reduce the excretion and dissemination of the virus and, therefore, the extent of an epizooty. While their efficacy against EHV-1-induced abortion in pregnant mares and the decreased occurrence of an abortion storm in the field have been reported, their potential efficacy against the neurological form of disease remains undocumented. No antiviral treatment against EHV-1 is marketed and recommended to date. This study aimed to measure the protection induced by valganciclovir (VGCV), the prodrug of ganciclovir, in Welsh mountain ponies experimentally infected with an EHV-1 ORF30-C_2254_ strain. Four ponies were administered VGCV immediately prior to experimental EHV-1 infection, while another four ponies received a placebo. The treatment consisted in 6.5 mg/kg body weight of valganciclovir administered orally three times the first day and twice daily for 13 days. Clinical signs of disease, virus shedding and viraemia were measured for up to 3 weeks. The severity of the cumulative clinical score was significantly reduced in the treated group when compared with the control group. Shedding of infectious EHV-1 was significantly reduced in the treated group when compared with the control group between Day + 1 (D + 1) and D + 12. Viraemia was significantly reduced in the treated group when compared with the control group. Seroconversion was measured in all the ponies included in the study, irrespective of the treatment received. Oral administration of valganciclovir induced no noticeable side effect but reduced clinical signs of disease, infectious virus shedding and viraemia in ponies experimentally infected with the EHV-1 C_2254_ variant.

## 1. Introduction

Equid alphaherpesvirus-1 (EHV-1) is one of the main pathogens of horses, with an estimated prevalence of 60% [1]. It is responsible for respiratory and ocular diseases, abortions, neonatal foal deaths and neurological signs such as equine herpesvirus myeloencephalopathy (EHM) [1,2]. The primary infection of EHV-1 targets the respiratory epithelial cells of the upper respiratory tract. Then, EHV-1 infects lymphocytes and subsequently reaches the blood circulation [3,4]. The cell-associated viraemia induces a dissemination of the virus to secondary sites of infection, such as the central nervous system (CNS), the uterus or the eye [5]. Several studies have suggested that EHM is associated with a single-nucleotide polymorphism at position 2254 in the EHV-1 DNA polymerase gene (ORF 30) [6,7]. Since these findings, EHV-1 G_2254_ (coding for an aspartic acid D_752_) strains are frequently labelled as neuropathogenic, and EHV-1 A_2254_ (coding for an asparagine N_752_) strains are considered to be non-neuropathogenic and usually associated with abortion and respiratory disease in horses [8]. However, the link between this mutation and the different forms of disease is still debated [9,10,11,12,13]. Recently, a new ORF30 variant of EHV-1 (C_2254_, coding for a histidine H_752_), was isolated in France and the USA and associated with respiratory and neurological diseases [14,15,16].

Vaccination has been part of the preventive strategy against EHV-1 for several decades. Current EHV vaccines reduce clinical signs of respiratory disease and virus shedding, which results in reduced EHV-1 transmission and, therefore, curtails the extent of an outbreak. While the frequency of EHV-1 abortion storms has dramatically decreased since the implementation of vaccination, current EHV-1 vaccines have limited claims against abortion, and efficacy against the neurological form of disease has not been reported [17]. To date, no EHV-1 antiviral treatment is commercialised or recommended. A recent study from our group has identified 21 compounds with in vitro antiviral activity against EHV-1 [18,19]. Among them, valaciclovir (VACV), the prodrug of aciclovir (ACV), is the only one that has been tested against EHV-1 in experimentally infected horses and ponies [20,21]. Garré et al. (2009) reported that the oral administration of 40 mg/kg of VACV, three times a day, for one week, did not show any beneficial effect after EHV-1 infection of young ponies [20]. Although the measured ACV plasma concentration was maintained in the range of the half-maximal effective concentration (EC_50_) previously determined in vitro (1.7–3.0 μg/mL) on equine embryonic lung cells [22]. In the study conducted by Maxwell et al. (2017), the oral administration of VACV (27 mg/kg, three times a day, for 2 days; followed by 18 mg/kg, two times a day, for 5 and 12 consecutive days) significantly reduced the virus shedding and the cell-associated viraemia in old mares experimentally infected with EHV-1 [21]. The overall cumulative clinical score was also reduced. However, the maximal measured plasma concentration of ACV was lower than the EC_50_ determined in vitro in the same study (11.4 ± 1.5 μg/mL) on equine foetal lung cells [21]. The discordance of these results shows the difficulty of transposing the in vitro data to in vivo. The administration of VACV was also reported during several natural outbreaks of EHV-1 in equids in the field [23,24,25,26]. However, the VACV treatment’s effectiveness was difficult to measure and/or confirm, notably due to a lack of controls.

Ganciclovir (GCV) is one of the most effective compounds identified against EHV-1 in vitro with an EC_50_ of 0.153 μg/mL on equine embryonic kidney (EEK) cells [18], which is 10-fold more active than ACV [22,27]. In addition, no difference of GCV susceptibly was observed between the three EHV-1 ORF30 variants [18]. However, the in vivo efficacy against EHV-1 of GCV, or its L-valyl ester prodrug valganciclovir (VGCV), has not been reported. Ganciclovir is a nucleoside analogue of 2′-deoxyguanosine, extensively used for the treatment of cytomegalovirus infections in humans [28,29]. Other human herpesviruses are also sensitive to GCV in vitro, including herpes simplex virus 1 and 2; varicella zoster virus; Epstein–Barr virus and human herpesviruses 6, 7 and 8 [30]. The oral bioavailability of GCV is poor in humans (7%) [31], which explains the preferential use of VGCV [32,33]. The bioavailability of GCV from oral VGCV administration is estimated to reach 60% in humans [34]. After oral administration, VGCV is well absorbed by the gastrointestinal tract and rapidly hydrolysed into GCV in the intestinal wall and liver. The increased bioavailability of VGCV seems related to its recognition as a substrate by the intestinal peptide transporter PEPT1 [35]. In horses, pharmacokinetics of GCV (intravenous route) and VGCV (oral route) were previously measured [36]. Oral administration of 1800 mg of VGCV dissolved in lemon juice and mixed with syrup and flour showed a mean GCV bioavailability of 41%. Although the oral absorption of VGCV is variable between horses, these elements support VGCV as a potential antiviral treatment against EHV-1. In addition, topical administration of GCV has recently been demonstrated to be effective in reducing the clinical signs of equine coital exanthema induced by EHV-3 [37].

Results from a randomised clinical trial in Welsh mountain ponies are reported here. The study aimed to measure the therapeutic efficacy of 2 weeks of VGCV treatment orally administrated immediately after experimental EHV-1 infection (FR- 56628; EHV-1 ORF30 C_2254_ variant). Ganciclovir pharmacokinetics, clinical signs of disease, viral shedding, cell-associated viraemia and seroconversion were measured for up to 3 weeks post infection.

## 2. Results

### 2.1. Valganciclovir Administration and Pharmacokinetics

Valganciclovir was dissolved overnight at 4 °C in commercial apple juice (pH = 3.22) for ease of administration to Welsh Mountain ponies, as previously described in lemon juice [36]. An in vitro experiment was first realised to determine any potential effect on VGCV activity linked to this method of preparation. VGCV tablets were dissolved either in apple juice or PBS (pH = 7.0) and stored at 4 °C overnight. Equine dermal fibroblasts (E. Derm, NBL-6 ATCC^®^ CCL-57^TM^, Manassas, VA, USA) were infected with EHV-1 Kentucky D (KyD) strain (ATCC^®^ VR-700^TM^) at an MOI of 0.01 and treated simultaneously with 10, 1 and 0.1 μg/mL of VGCV (dissolved in apple juice and PBS) and monitored for 72 h, as previously described [18]. Results showed that apple juice did not reduce the antiviral effect of VGCV at 1 and 0.1 μg/mL, when compared to VGCV dissolved in PBS (Figure S1). At 10 μg/mL, EHV-1 in vitro infection has no significant effect on the cell proliferation when compared with the mock infected cells.

**Figure 1:**
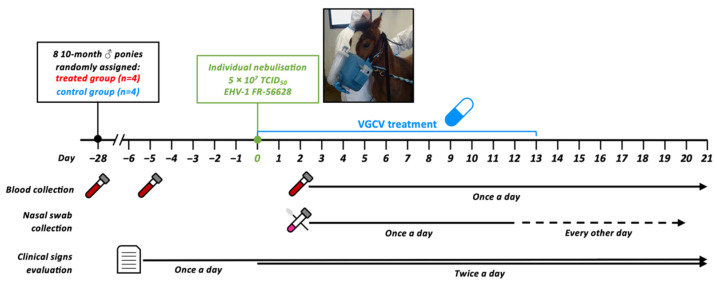
Study design. Eight ponies were placed together in the same stable and were infected by individual nebulisation with a total of 5 × 10^7^ tissue culture infectious dose 50 (TCID_50_) of EHV-1 FR-56628 strain. Ponies in the treated group (ponies A, B, C and D) were treated for 2 weeks starting from the first day of the challenge (D0 to D + 13), whereas the control group (ponies E, F, G and H) received a placebo. Nasal swabs, blood collection and clinical signs were evaluated for the duration of the challenge. All ponies were euthanised at D + 21.

**Figure 2:**
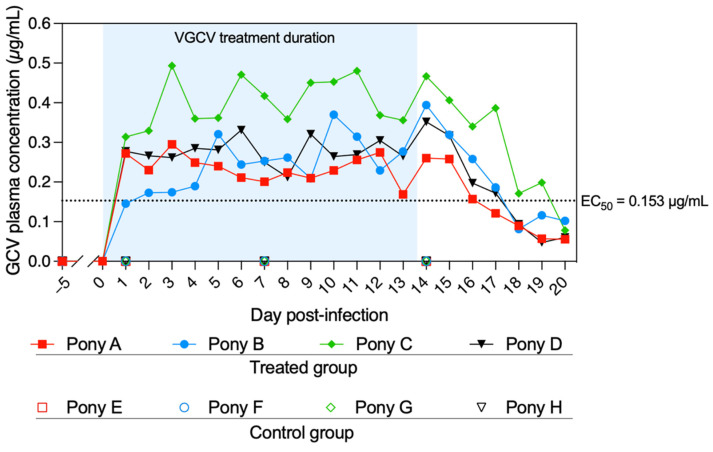
Serum concentration of ganciclovir (GCV) from D − 5 to D + 20. Ponies A to D (treated group) were administered valganciclovir (VGCV) from D0 to D + 13, and ponies E to H (control group) received a placebo. Blood samples were taken just prior to the first daily VGCV administration. No GCV was detected in the sera of the control group ponies at D − 5, D + 1, D + 7 and D + 14. The half-maximal effective concentration (EC_50_) of GCV was previously determined in vitro against the EHV-1 FR-56628 strain on equine embryonic kidney (EEK) cells [18]. The blue shading represents the duration of VGCV treatment.

Valganciclovir was administered to all treated ponies from Day 0 (D0) to Day +13 (D + 13) and sera were collected at D − 28, D − 5 and from D + 2 to D + 21, as presented in Figure 1. The concentration of GCV was measured in the sera collected from the treated and untreated ponies at different time points (Figure 2). Prior to treatment (D0), no GCV was detected in any of the ponies’ serum. GCV was detected in all sera from treated ponies from D + 1 to D + 20. GCV serum concentration was maintained above the EC_50_ of 0.153 µg/mL previously determined in vitro against the EHV-1 FR-56628 (C_2254_) strain [18] in all treated ponies for the duration of treatment (D + 1 to D + 13, mean concentration = 0.295 ± 0.068 µg/mL). At D + 20 (7 days after the end of the treatment), GCV was still detectable in the blood of all treated ponies (0.074 ± 0.021 µg/mL). All control ponies remained negative when tested on days D + 1, D + 7 and D + 14.

### 2.2. Clinical Signs of Disease

All ponies developed clinical signs of disease after experimental infection with EHV-1 FR-56628 strain (fever, nasal discharge, cough, mandibular lymphadenopathy, ocular discharge and behaviour). The number of observations and the severity of the clinical signs of disease are presented in Table 1.

**Figure 3:**
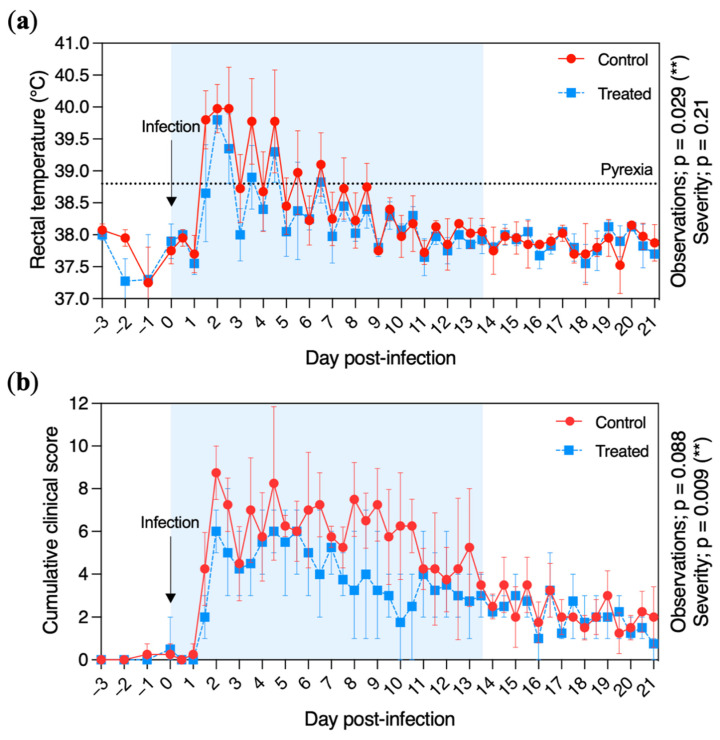
Clinical signs of disease recorded from the control (red circles and solid line) and treated (blue squares and dotted line) groups. (**a**) Daily rectal temperature (°C) of ponies before and after experimental infection with EHV-1 FR-56628 strain (D0). Temperatures >38.8 °C were considered pyretic. (**b**) Cumulative clinical score before and after experimental infection. The cumulative clinical score was calculated using the score for each clinical sign according to the formula described in Materials and Methods. Rectal temperature and clinical evaluations were taken and performed daily from day 3 prior to EHV-1 infection (D − 3) to D − 1 and twice a day from the day of infection (D0) to 21 days post infection (D + 21). The mean and standard deviation per group is shown for each time point. The blue shading of each graph represents the duration of VGCV treatment.

The peak of pyrexia was reached at D + 2 for both the control and treated groups (Figure 3a). Pyrexia was recorded in the control group from D + 1 to D + 8 included (with an average of 15 observations per pony), while treated ponies were pyretic for 6.8 ± 2.6 observations from D + 1 to D + 6 included. The average number of fever observations was significantly reduced in the treated group when compared with the control group (*p* = 0.029). However, the severity (based on the body temperature measured in °C) was similar between the treated and the control groups, with a maximum rectal temperature of 39.8 and 40.0 °C, respectively, measured at D + 2.

Nasal discharges were observed from D + 1 and recorded throughout the study. The duration and the severity of nasal discharges were significantly reduced in the treated group when compared with the control group (*p* = 0.033 and *p* = 0.028, respectively). All control ponies showed mucopurulent discharges for 3 to 7 days between D + 3 to D + 12. Mucopurulent discharges were observed for three treated ponies—at D + 5 for ponies A and D and D + 7 for pony B—and no mucopurulent discharges were observed for pony C.

Cough was recorded for one control pony at D + 1 (pony E) and in all control ponies and only two treated ponies at D + 2 (ponies A and C). Cough was then recorded intermittently from D + 3 to D + 21 in all ponies. The cough severity was significantly reduced in the treated group (*p* = 0.0012). However, the number of observations of cough was not significantly different between the two groups.

Mandibular lymph node enlargement was observed for one control pony on D + 2 (pony G), and in all control ponies from D + 3 to D + 11. Mandibular lymph node enlargement was observed on D + 3 for two treated ponies (A and D) and on D + 4 for two others (B and C). Mandibular lymph nodes returned to their normal size at D + 6.5 for pony B and at D + 14 for the three other treated ponies.

All ponies from the control group and three ponies from the treated groups (A, B and C) showed ocular discharges with a peak at D + 3 and D + 4.

All ponies showed signs of lethargy on D + 2 and had a tail hypotonia from D + 2 to D + 4/5. Reduction of food consumption was also observed during the first week post infection. No pony showed sign of neurological disease during this study.

The daily cumulative clinical score is shown in Figure 3b. It increased rapidly after infection, with a peak at D + 2 (8.8 ± 1.6) for the control group and a peak at D + 4.5 (6.3 ± 1.0) for the treated group. The daily cumulative clinical score decreased at D + 7 and D + 10 for the treated and control groups, respectively. The cumulative clinical score severity was significantly reduced in the treated group when compared with the control group (*p* = 0.0088).

### 2.3. Nasopharyngeal Shedding

**Figure 4:**
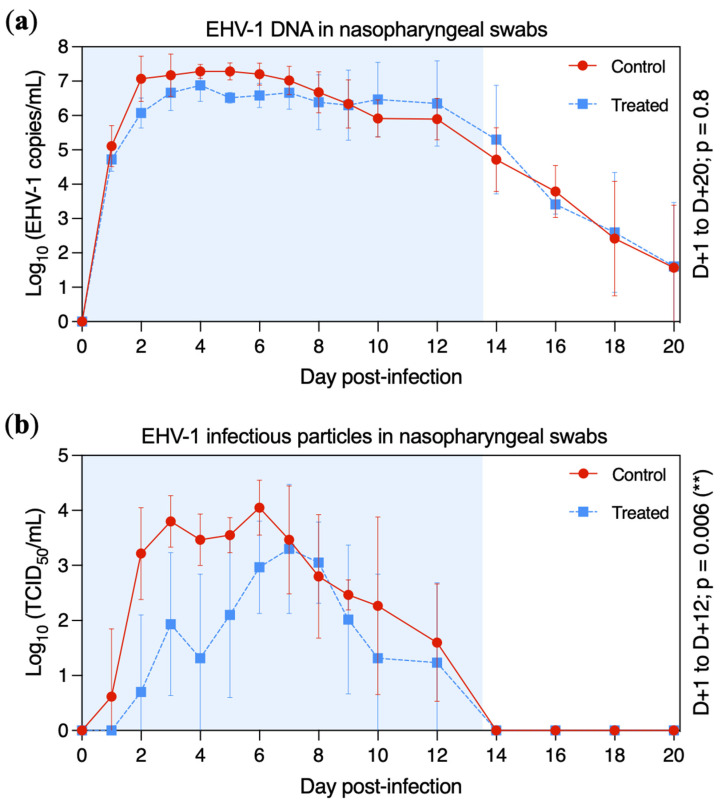
Mean ± standard deviation of EHV-1 DNA and infectious particles detected in nasopharyngeal swabs in the control group (red circles and solid line) and the treated group (blue squares and dotted line). All ponies were inoculated with EHV-1 FR-56628 strain on day 0 (D0). Nasopharyngeal shedding was measured by qPCR assay (**a**) and infectious virus titres were measured on an RK13 cell monolayer (**b**). The blue shading of each graph represents the duration of VGCV treatment.

EHV-1 DNA was detected by quantitative PCR (qPCR) in nasopharyngeal swab extracts from all ponies from D + 1 to D + 16 (Figure 4a). At D + 18, EHV-1 DNA was detected in three ponies from each group (treated ponies: A, B and C; control ponies: F, G and H). At D + 20 (last sample collected) EHV-1 DNA was still detectable in only two ponies per group (treated ponies: B and C; control ponies: F and G). The highest quantity of EHV-1 DNA was measured at D + 4 for both groups, with a plateau phase between D + 2 and D + 12. From D + 14, the amount of EHV-1 DNA in the nasopharyngeal swab extracts drastically decreased for both groups. The amount of viral DNA was not significantly different between treated and control groups from D + 1 to D + 20 (*p* = 0.8), when measured by qPCR assays. 

Titration of infectious viral particles in nasopharyngeal swab extracts was measured on normal rabbit kidney epithelial (RK13, ATCC^®^, Manassas, VA, USA) cells (Figure 4b). Infectious virus was isolated from one control pony at D + 1 (pony H) and in all four control ponies at D + 2. EHV-1 was isolated from one treated pony at D + 2 (pony A), three treated ponies at D + 3 (ponies A, C and D) and in all treated ponies at D + 6. The highest titre was measured at D + 6 for the control group (4.05 ± 0.50 tissue culture infectious dose 50 (TCID_50_)/mL) and at D + 7 for the treated group (3.30 ± 1.17 TCID_50_/mL). Infectious virus titres began to decrease at D + 6 and D + 7 (control and treated groups, respectively). From D + 14, no infectious EHV-1 was detected in any ponies. Infectious virus shedding was significantly reduced in the treated group when compared with the control group between D + 1 to D + 12 (*p* = 0.006).

### 2.4. Viraemia

**Figure 5:**
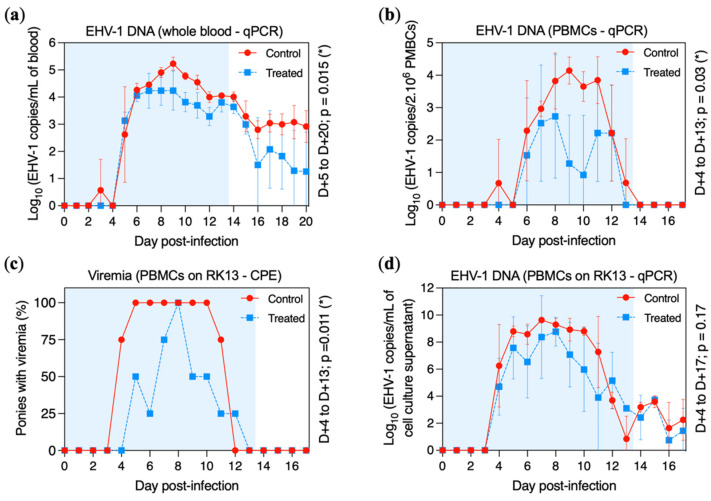
Viral DNA and infectious particle detection in the blood of the control group (red circles and solid line) and the treated group (blue squares and dotted line). All ponies were experimentally infected with EHV-1 FR-56628 strain at day 0 (D0). Presence of EHV-1 DNA was measured by (**a**) qPCR assay in 2 mL of blood or (**b**) in 2 × 10^6^ PBMCs. (**c**) Percentage of ponies with cell-associated viraemia when 1.10^6^ PBMCs were co-cultured during 72 h on an RK13 cell monolayer and observed by microscopy. (**d**) Presence of EHV-1 DNA measured by qPCR assay in the cell culture lysate collected from these co-cultures (i.e., 1 × 10^6^ PBMCs co-cultured during 5 days on an RK13 cell monolayer). The blue shading of each graph represents the duration of VGCV treatment.

EHV-1 DNA was detected in all ponies when viral DNA extraction was performed on 2 mL of whole blood (Figure 5a). Viral DNA was detected in one control pony at D + 3 (pony E) and in all control ponies from D + 5 to D + 20 with a peak at D + 9 (5.23 ± 0.24 Log_10_ EHV-1 copies/mL). Viral DNA was detected in all treated ponies from D + 5 to D + 15 and intermittently from D + 16 to D + 20. Viraemia was significantly reduced in the treated group when compared with the control group between D + 5 to D + 20 (*p* = 0.015).

When DNA extraction was performed on 2 × 10^6^ peripheral blood mononuclear cells (PBMCs), cell-associated viraemia was detected in one control pony at D + 4 (pony H), in all control ponies from D + 6 to D + 11, in three control ponies at D + 12 (ponies F, G and H) and in only one control pony at D + 13 (pony G) (Figure 5b). Viral DNA was detected in two treated ponies at D + 6 (ponies A and C) and in three treated ponies from D + 7 to D + 12 (ponies A, B and D), but only one pony showed cell-associated viraemia at D + 10 (pony D). The peak of cell-associated viraemia was reached at D + 9 (4.14 ± 0.42 Log_10_ EHV-1 copies/2 × 10^6^ PBMCs) and at D + 8 (2.73 ± 1.91 Log_10_ copies/2 × 10^6^ PBMCs) in the control and treated groups, respectively. Cell-associated viraemia was significantly reduced in the treated group when compared with the control group between D + 4 to D + 13 (*p* = 0.03).

Cell-associated viraemia was also measured by co-culture of 1 × 10^6^ PBMCs on an RK13 cell monolayer for 72 h. Results of cytopathic effect (CPE) formation and viral DNA quantitation in cell culture lysate are presented in Figure 5c and Figure 5d, respectively. CPEs were observed from D + 4 to D + 11 for the control group and from D + 5 to D + 12 for the treated group. The number of positive samples was significantly reduced in the treated group when compared with the control group (*p* = 0.011). When viral DNA was measured by qPCR assay, EHV-1 was detected from D + 4 to D + 17 in both groups. No significant difference was observed between the treated and control groups from D + 4 to D + 17 (*p* = 0.17; Figure 5d). 

### 2.5. Pathogen Identification

The ORF30 C_2254_ mutation was confirmed in nasopharyngeal and blood samples collected at D + 5, D + 10, D + 16 and D + 20 from all ponies. Neither EHV-1 strains with ORF30 A_2254_ and G_2254_ mutation nor the EHV-4 strains were detected in any samples (Table S1). 

### 2.6. Serological Response

**Figure 6:**
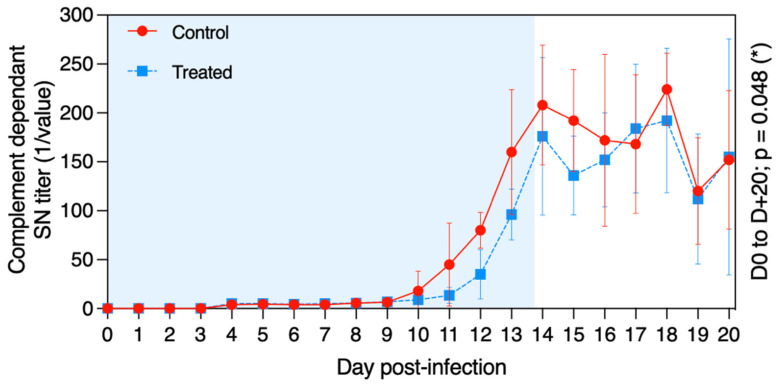
Mean ± standard deviation of serum neutralisation (SN) antibody titre against EHV-1/4 in samples collected from each pony in the control group (red circles and solid line) and in the treated group (blue squares and dotted line) after experimental infection with EHV-1 FR-56628 strain at D0. The blue shading of each graph represents the duration of VGCV treatment.

The virus neutralisation antibody titre was measured at D − 5 and each day from D + 0 to D + 20 in the serum of all ponies (Figure 6). Seroconversion was detected from D + 10 to D + 14 for all ponies included in this study. When considering the whole period (D0 to D + 20), serum neutralisation (SN) titres were significantly higher in the control group when compared with the treated group (*p* = 0.048). This was particularly noteworthy before D + 16.

## 3. Discussion

This study investigates the antiviral effect of valganciclovir (VGCV) treatment, the oral prodrug of ganciclovir (GCV), on Welsh mountain ponies experimentally infected with an EHV-1 ORF30 C_2254_ strain (FR-56628). Ponies were monitored for 4 weeks before challenge to confirm their EHV-1/4-naive status prior to infection: (1) no clinical sign of infection was recorded, (2) no virus (EHV-1, 4 and equine influenza virus) was detected in nasopharyngeal secretion and (3) none of them had detectable EHV-1 neutralising antibodies during this pre-inoculation period. In addition, seroconversion was measured between D + 10 and D + 14 after experimental infection, which is typical of a primary serological response to EHV-1 infection when measured in naive horses [38]. 

No adverse effect was observed throughout this study in any ponies after the oral administration of VGCV. This result agrees with a previous study where a single oral dose of VGCV was administrated in horses with no noticeable side effect [36]. In the same study, the GCV serum concentration (pharmacologically active drug) peaked less than 2 h after the oral administration of a single dose of VGCV (1800 mg) [36]. Based on this result, the first dose of VGCV was given 2 h before infection in the present study. The GCV serum concentration was maintained for the duration of the treatment (i.e., 14 days) above the EC_50_ value (0.153 µg/mL), which was previously determined in vitro on equine embryonic kidney cells [18]. This demonstrated that the dose of VGCV and the administration interval was appropriate in this study. Furthermore, according to the supplier’s instructions, it should be noted that GCV is a potential teratogen and carcinogen in humans. Even though no observed teratogenic effects were observed in human case reports [39,40], the use of VGCV treatment in pregnant mares should be considered with caution.

Ponies were experimentally infected with the EHV-1 ORF30 C_2254_ (FR-56628) strain [14] by individual nebulisation, as previously described for equine influenza [41]. This strain was previously isolated from equine PBMCs during an outbreak occurring in France in 2018. During this outbreak, numerous cases of respiratory disease and two cases of EHM were recorded, leading to the euthanasia of one of them [15]. In the present study, all infected ponies developed respiratory signs two days post infection. Ocular discharges, submandibular lymph node enlargement and loss of tail tone were also observed. However, no neurological signs associated with EHV-1 infection were recorded during this study. It has been previously reported that old horses and mares were more susceptible to develop neurological signs after EHV-1 infection [42,43,44,45], while ponies were less susceptible [2,45,46]. This could explain the absence of neurological signs in the current study (i.e., all ponies were 10-month-old males). 

Fever and respiratory signs of disease (nasal discharge and cough) induced by EHV-1 were significantly reduced in the VGCV-treated group when compared with the placebo group. The antiviral effect of another anti-herpetic molecule, valaciclovir (VACV; the prodrug of acyclovir), was previously measured in ponies [20] and horses [21] during experimental infections with EHV-1. In the first study, no beneficial effects were observed after VACV treatment [20]. In contrast, pyrexia, cumulative clinical signs, nasopharyngeal discharge and viraemia were reduced when VACV was administered to horses one day before virus inoculation [21].

In this present study, virus excretion was measured with two different methods (qPCR and virus titration in cell culture). EHV-1 was detected by qPCR from D + 1 to D + 16 in the nasopharyngeal secretions of all ponies. Viral DNA shedding lasted up to 20 days for some individuals. All nasopharyngeal swab extracts that tested positive by qPCR assay (qPCR+) were analysed by cell culture. EHV-1 could not be isolated on cell culture after D + 12. Only 57% (qPCR+ samples from controls) and 41% (qPCR+ samples from treated ponies) were found positive by cell culture. Virus isolation on cell culture allowed the detection of infectious particles in nasopharyngeal swab extracts but was less sensitive when compared to the qPCR test, as previously described [47]. As no EHV-1 was isolated from D + 14, which also corresponds to the peak virus neutralisation antibody titre, subsequent EHV-1 qPCR detection was probably associated with non-infectious viral particles. In our study, the antiviral treatment with VGCV slightly delayed the peak of infectious virus excretion (cell culture) and significantly reduced the amount of infectious virus released in nasopharyngeal discharges. However, this was not measured with qPCR. While encouraging, it is not possible to know whether the VGCV antiviral effect measured in this study could reduce virus transmission between individuals.

Secondary bacterial complication is usually observed after respiratory virus infection but could be minimised in vaccinated horses, as previously described after equine influenza vaccination and experimental infection [48]. In this study, mucopurulent discharges were observed in all control ponies from D + 4 to D + 12 but only for one day between D + 4 to D + 9 in three ponies in the treated group. This result tends to indicate that the limitation of EHV-1 infection by VGCV could also reduce the risk of secondary bacterial infection. 

Viraemia is known to be an important factor involved in the development of secondary forms of the disease, such as abortion and EHM [2,5]. The presence of EHV-1 in the blood was measured using different methods, EHV-1 DNA quantitation from whole blood, viral DNA quantitation from 2 × 10^6^ PBMCs and co-culture of 1 × 10^6^ PBMCs on an RK13 monolayer. Although all treated ponies exhibited viraemia, it was significantly reduced when compared with the control group. Viraemia appears on D + 3/D + 4 post infection, according to the methods listed above, which corresponds to 2–3 days after the onset of clinical signs of disease, in agreement with previous studies [38,49]. This gap between onset of clinical signs and viraemia provides an opportunity to start antiviral treatment in order to reduce the intensity and/or duration of cell-mediated viraemia and the subsequent risk of secondary forms of disease. When viral DNA quantitation was made from 2 mL of blood, EHV-1 was found in all the control ponies and in two treated ponies at the last collection day (D + 20). In contrast, when viral DNA quantitation was directly made from 2 × 10^6^ PBMCs, no viral DNA was found from D + 13 and D + 14 in treated and control groups, respectively, which corresponds to the establishment of the serological response. With the absence of infectious EHV-1 particles detected after D + 12/D + 13, results of EHV-1 co-culture are correlated to the viral quantitation made from 2 × 10^6^ PBMCs. Viral DNA quantitation is more sensitive in whole blood. The difference observed with quantitation on PBMCs could be partially explained by the cell purification process. It could also be explained by the low frequency of infected PBMCs after D + 14. However, the co-culture experiment allowed the detection of viral DNA after D + 14 (viral particles were intermittently measured in cell culture lysate after D + 13 in both groups of ponies, although no CPE was observed at these time points). Our results suggest that an absence of CPEs does not mean that there is no infectious virus in the sample and underlines the importance of the limit of detection of the techniques used [50,51]. Considering the different methods used, the result clearly demonstrated that VGCV treatment reduced the viraemia and, thus, the risk of secondary forms of the disease. In addition, the establishment of latency in the trigeminal ganglia and respiratory associated lymphoid tissue is an important part of EHV-1 pathogenicity. However, this study did not evaluate whether VGCV treatment influenced latency establishment. Furthermore, it would be interesting to evaluate whether VGCV treatment prevents reactivation of the latent virus.

VGCV was previously demonstrated to be effective at comparable concentrations in vitro against numerous EHV-1 field strains including the three ORF30 variants (A_2254_, C_2254_, G_2254_) [18]. It could be supposed that the VGCV antiviral activity measured in this study against the EHV-1 C_2254_ strain is likely to apply to other EHV-1 strains. Further studies should be carried out to optimise the therapeutic protocol. The antiviral effect observed in the current study could be improved in the future by (1) increasing the amount of VGCV administered and (2) by combining the VGCV treatment with another antiviral compound. Our group has recently demonstrated that VGCV acts synergistically with decitabine, a deoxycytidine analogue, in vitro against EHV-1 [18]. However, further in vitro and in vivo experiments are required before conducting an experimental infection with these two compounds. In conclusion, the oral administration of VGCV significantly reduced clinical signs of disease and infectious virus shedding in Welsh mountain ponies experimentally infected with EHV-1. In complement to the current prevention and management measures, such results highlight the potential of VGCV as a strategy to limit the development of secondary forms of disease during EHV-1 outbreaks. 

## 4. Materials and Methods

### 4.1. Animals and Study Design

Eight 10-month-old males Welsh Mountain ponies were included in this study. They were weighed at 41 (D − 41) and 10 days (D − 10) before experimental infection. They were between 124 and 162 kg (median at 153 kg) of body weight. Ponies were randomly assigned to the treated or control group of four subjects (http://www.jerrydallal.com/random/randomize.htm; seed 28092; accessed on 18 February 2020). All ponies were seronegative for EHV-1/4 as determined by seroneutralisation and complement fixation tests at D − 28 and D − 5. qPCR detection in nasal secretions for EHV-1, EHV-4, EHV-2, EHV-5 and equine influenza virus were all negative at D − 5. All animals were housed together in an isolated and daily cleaned stable. They were fed daily with a commercial complete feed, and drinking water was supplied ad libitum. All experiments were conducted in accordance with the guidelines of the Directive 2010/63/EU of the European Parliament and of the Council, in the facilities of the Infectiology of Farm, Model and Wildlife Animals Facility (PFIE, Centre INRAE Val De Loire; member of the National Infrastructure EMERG’IN). All experimental procedures were approved by the Loire Valley ethical review board (CEEA VdL, committee number 19, authorisation number APAFiS#22708, 8 January 2020). All laboratory experiments were performed under blind conditions. Due to the management, blinding was not possible for clinical evaluation in this study. Experimental design of this study is depicted in Figure 1. This report follows the CONSORT 2010 guidelines (Figures S2 and S3) [52,53].

### 4.2. Virus and Experimental Infection

The French EHV-1 strain FR-56628 was used after 3 passages on RK13 cells. This strain was isolated in 2018 from the PBMCs of one horse showing respiratory diseases and carries a cytosine mutation at position 2254 in the viral DNA polymerase gene (ORF30) [14,15].

Individual nebulisation was performed using a foal Flexineb^®^ nebuliser (VLC Europe, Bazoches sur Guyonne, France; Figure 1) at day 0 (D0), as previously described for equine influenza virus experimental infection [41]. The inoculum, containing 2 mL of virus suspension holding 5 × 10^7^ TCID_50_, was placed in the holding chamber of the nebuliser attached to a face mask. The duration of nebulisation was 2 min. The inoculum concentration was checked on RK13 cells before nebulisation using the Spearman–Kärber method [54].

### 4.3. Valganciclovir Administration

Four of the eight ponies were treated with VGCV (treated group), while the other four received placebo (control group). The treatment consisted in 6.5 mg/kg body weight of valganciclovir (Mylan S.A.S., Saint-Priest, France) three times the first day (D0) and twice daily for 13 days (D + 1 to D + 13). The first dose was given 2 h prior to inoculation. The treatments were prepared identically the day before use: 450 mg VGCV tablets (Valcyte^®^, Roche, Bale, Switzerland) were dissolved in apple juice at 100 mg/mL and stored overnight at 4 °C. The appropriate volume of VGCV solution, depending on the weight of the animal, was mixed just before treatment in a drug gun containing 15 mL of apple compote (Figure S4). The placebo treatment contained 10 mL of apple juice mixed with 15 mL of apple compote. Different drug guns were used for the two groups, and all treatments were given as a reward after clinical observations and sample collection.

### 4.4. Clinical Observation

For all ponies, clinical signs and rectal temperature were recorded once a day from D − 6 to D − 1 and twice a day from D0 to D + 21. A rectal temperature (RT) of >38.8 °C was regarded as fever. Behaviour (BV), nasal discharges (ND), coughing (CG), ocular discharges (OD), lymph node swelling (LN) and nervous disorders (ND) were monitored during each clinical examination. The order of passage of the ponies for the latter was random. A daily clinical score for each pony was defined using the scoring presented in Table S2. The cumulative clinical score (CCS) was calculated each day using a formula adapted from the score for equine influenza virus infection in Welsh mountain ponies [55]:

CCS = RT + ND + 2 × (CG) + LN + OD + BV + ND

### 4.5. Blood Collection and Processing

Blood samples were collected once a day by jugular venepuncture into EDTA (10 mL) and dry tubes (10 mL) at D − 5 and from D + 1 to D + 20 just before treatment administration. Collected sera in dry tubes were frozen at −20 °C until use. Following this, 2 mL of EDTA-treated blood sample was used for viral DNA extraction, as described in Section 4.8. PBMCs were isolated from the remains of the EDTA tubes by density centrifugation using lymphocyte separation medium (Eurobio, Courtaboeuf, France). Cells were rinsed three times with phosphonate-buffered saline (Eurobio) and suspended in RPMI medium (Eurobio) supplemented with 10% foetal bovine serum (Eurobio), 100 IU/mL penicillin, 0.1 mg/mL streptomycin and 0.25 μg/mL amphotericin B (Eurobio). PBMCs were counted using an inverted microscope. In all, 2 × 10^6^ PBMCs were collected and frozen at −20 °C for viral quantitation, and 1 × 10^6^ PBMCs were collected and used for co-culture on RK13 monolayer cells.

### 4.6. Nasal Swab Collection and Processing

Nasopharyngeal samples were collected at D − 5, each day from D + 1 to D + 10 and at D + 12, D + 14, D + 16, D + 18 and D + 20 using 745 mm sterile swabs for mares (IMV Technologies, L’Aigle, France). Swabs were inserted through the nostrils up to the nasopharynx, alternating the left and right nostril each day. Swabs were then placed directly into polypropylene tubes containing 3 mL of minimum essential medium (MEM) with Earle’s salts (Eurobio) complemented with 1% L-glutamine (Eurobio), 100 IU/mL penicillin, 0.1 mg/mL streptomycin and 0.25 μg/mL amphotericin B (Eurobio). Nasopharyngeal swabs were then vortexed and centrifuged for clarification, and the supernatant was frozen at −20 °C for viral titre determination and viral quantitation.

### 4.7. Viral Titre Determination and Co-Culture

RK13 cells were cultivated in MEM with Earle’s salts (Eurobio) complemented with 10% foetal bovine serum (Eurobio), 1% L-glutamine (Eurobio), 100 IU/mL penicillin, 0.1 mg/mL streptomycin and 0.25 μg/mL amphotericin B (Eurobio) at 37 °C and 5% CO_2_. Subsequently, 1 × 10^6^ PBMCs were seeded on RK13 monolayer cells on 6-well plates and incubated at 37 °C for 72 h. The presence of viral cytopathic effects was observed using an inverted microscope, and plates were frozen at −80 °C for viral quantitation. Eight tenfold serial dilutions of swab samples were inoculated in triplicate on RK13 monolayer cells on 96-well plates and incubated at 37 °C for 7 days. Wells with cytopathic effects were recorded, and the TCID_50_ of each sample was determined using the Spearman–Kärber method [54].

### 4.8. Viral DNA Extraction and Quantitation

Viral DNA was extracted from the EDTA-treated blood sample using a Nucleospin^®^ Blood L kit (Macherey-Nagel, Hoerdt, France) according to the manufacturer’s instructions. Viral DNA was extracted from 2 × 10^6^ PBMCs, 140 µL of viral inoculum stock, 140 µL of clarified nasopharyngeal swabs and 140 µL of cell culture lysate using QIAamp^®^ Viral RNA Mini Kit (Qiagen, Courtaboeuf, France) according to the manufacturer’s instructions. EHV-1 ORF30 C_2254_ mutation in the viral inoculum stock and absence of EHV-1 strains with G_2254_ and A_2254_ mutations, EHV-4, EHV-2, EHV-5 and equine influenza virus was confirmed by qPCR before infection. qPCR for EHV-1, EHV-4, EHV-2, EHV-5 and equine influenza virus was performed as previously described [56]. Typing EHV-1 qPCR (G_2254_/A_2254_/C_2254_) was performed as described previously [15]. Each reaction was performed in a total volume of 25 µL containing 12.5 µL of 2× Taqman^®^ Universal Master Mix (Life Technologies, Villebon-surYvette, France), 1.25 µL of primers, 1.5 µL of probes, 6 µL of nuclease-free water and 2.5 µL of template DNA using QuantStudio^TM^ 12 K Flex Real-Time PCR System (Life Technologies). The limit of detection (LOD 95%) for EHV-1 qPCR [57] is 7.2 copies/µL of reaction. Below this threshold, the accuracy of signal quantification is reduced. Sequences of primers, probes and thermal cycling are described in Table S3. 

### 4.9. Serological Analysis

The neutralising antibody assay was performed using the sera of all 8 ponies collected at D − 5 and D + 1 to D + 20 according to the OIE method [58]. Briefly, seven twofold serial dilutions of each serum from 1:4 to 1:256 were performed in duplicate on 96-well plates using complemented MEM with Earle’s salts (Eurobio). The EHV-1 strain KyD (ATCC^®^ VR-700^TM^) was added to each dilution at a concentration of 100 TCID_50_/well, and plates were incubated at 37 °C for one hour. Virus and sera mixtures were then transferred on RK13 monolayer cells seeded in 96-well plates and incubated at 37 °C and 5% CO_2_. After 3 days of culture, cells monolayers were examined using an inverted microscope. The SN titre was estimated as the last dilution of serum neutralising the virus (no cytopathic effect formation). 

### 4.10. Ganciclovir Quantitation

GCV concentration was determined in sera at D − 5 and at D0 to D + 20. After a freezing period at −20 °C, 200 µL of sera were added to 600 µL of the internal standard in acetonitrile and were then centrifuged at 5000 rpm for 30 min. Next, 500 µL of supernatant was recovered and evaporated using EZ-2 series GENEVAC during 150 min. Then, 100 µL of acetonitrile with 0.1% of formic acid was added in dry tubes, and 400 µL of high-purity water with 0.1% of formic acid was added. Tubes were then incubated at room temperature for 30 min and filtered using a 0.45 µm polyvinylidene fluoride filter. The ganciclovir concentration was determined using UHPLC-MS/MS 6495 (Agilent Technologies) with Acquity HSS C18 column and Acquity HSS C18 VANGUARD pre-column (Waters corporation). A standard curve from 0.025 to 1 µg/mL was used for GCV quantification. Deuterium-marked ganciclovir (Toronto Research Chemicals) was used as internal standard for the quantification of GCV. 

### 4.11. Statistical Analysis

Statistical analyses were performed on StatGraphics^®^ Centurion XVI Version 16.1.12 for windows (StatPoint Technologies, Inc., Warrenton, VA, USA). The duration and severity of each clinical sign were compared between the treated and control groups using Student t-test from D0 to D + 21 (normality was confirmed using the standardised skewness and kurtosis). Statistical analysis for nasopharyngeal shedding, viraemia and seroneutralisation were evaluated using multifactor ANOVA test. Data were considered significant at a *p*-value < 0.05. 

## Figures and Tables

**Table 1:** Means ± standard deviation (S.D.) of the number of clinical observation (observations were performed twice a day) and severity of signs of disease for the control and treated groups (4 ponies by group) from D − 3 before infection to D + 21 post infection (* *p* < 0.05, ** *p* < 0.01; normal distribution, Student *t*-test).

Clinical Signs	Type of Analysis	Control Group (Mean ± S.D.)	Treated Group (Mean ± S.D.)	*p*-Value
Pyrexia (>38.8 °C)	Observation (n)	15.0 ± 3.6	6.8 ± 2.6	0.029 (*)
	Severity	1.9 ± 0.5	1.0 ± 0.2	0.21
Nasal discharge	Observation (n)	39.3 ± 1.5	36.0 ± 1.8	0.033 (*)
	Severity	2.5 ± 0.4	1.9 ± 0.1	0.028 (*)
Cough	Observation (n)	9.5 ± 5.9	5.5 ± 4.7	0.56
	Severity	1.3 ± 0.1	1.0 ± 0.1	0.0012 (**)
Mandibular lymph node size	Observation (n)	23.8 ± 4.0	22.3 ± 11.6	0.56
	Severity	1.2 ± 0.1	1.4 ± 0.3	0.33
Ocular discharge	Observation (n)	9.8 ± 9.0	6.5 ± 5.5	0.96
	Severity	1.2 ± 0.3	1.2 ± 0.8	0.56
Behaviour	Observation (n)	2.0 ± 0.0	2.0 ± 0.0	N.D.
	Severity	1.0 ± 0.0	1.0 ± 0.0	N.D.
Cumulative clinical score	Observation (n)	40.5 ± 0.6	39.3 ± 1.0	0.088
	Severity	4.5 ± 0.4	3.3 ± 0.5	0.0086 (**)

## Supplementary Materials

The following supporting information can be downloaded at https://www.mdpi.com/article/10.3390/pathogens11050539/s1: Figure S1: Real-time monitoring of E. Derm cells infected or uninfected by EHV-1 KyD strain and treated with valganciclovir (VGCV) dissolved overnight in PBS or apple juice; Figure S2: CONSORT checklist; Figure S3: CONSORT flow diagram; Figure S4: Drug gun loaded with apple compote and apple juice ± valganciclovir; Table S1: Determination of the EHV-1 strains and EHV-4 found in nasopharyngeal swabs and blood of ponies at different times post infection; Table S2: Description of the clinical sign score; Table S3: Amplification conditions for virus detection by qPCR.
