# Supplementary material for: Oral Administration of Valganciclovir Reduces Clinical Signs, Virus Shedding and Cell-Associated Viremia in Ponies Experimentally Infected with the Equid Herpesvirus-1 C2254 Variant
Source: Pathogens. 2022 May 4;11(5):539. doi: 10.3390/pathogens11050539 (PMC9148010; doi:10.3390/pathogens11050539)
Supplement: Supplementary file 1 [file pathogens-11-00539-s001.zip › Figure S1.pdf]

(a)

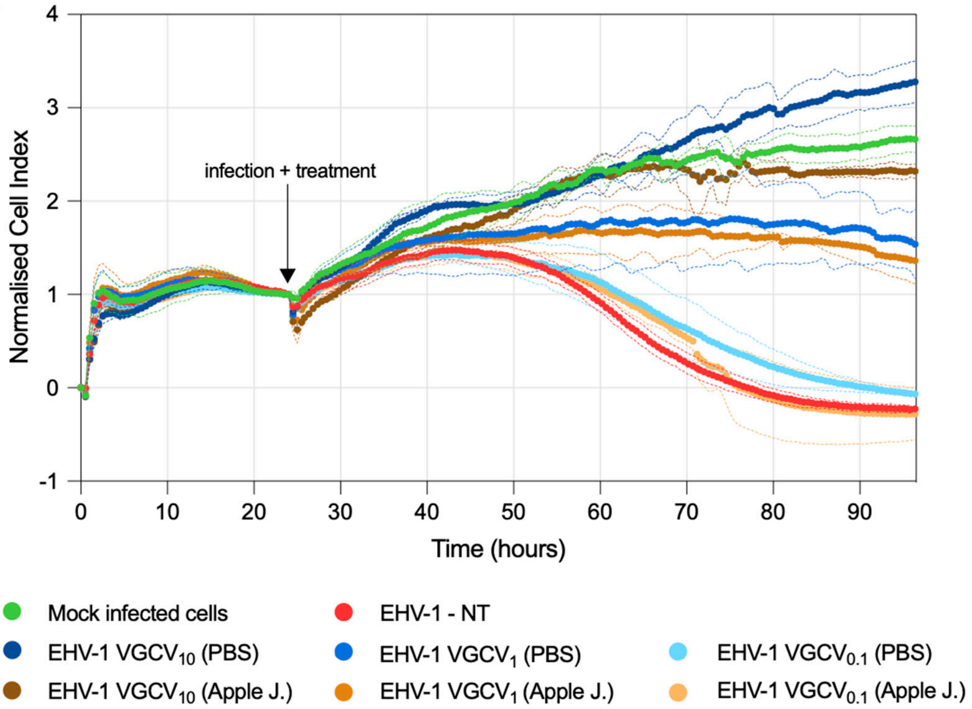

(b)

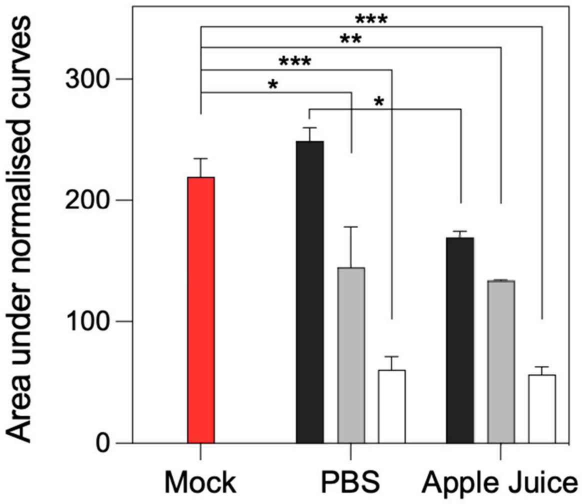

Figure S1. (**a**) Real time monitoring of E. Derm cells infected or not by EHV-1 KyD strain and treated with valganciclovir (VGCV) dissolved over-night at 4°C in PBS or apple juice. The Cell Index was normalized (CI_n) at the last time point before infection (black arrow). The red curve represents the CI_n of untreated EHV-1 infected cells (NT). The green curve represents the CI_n of mock infected cells. Blue and orange shade curves represent the CI_n of EHV-1 infected cells and treated with VGCV at 10 µg/mL ($VGCV_{10}$), 1 µg/mL ($VGCV_1$) and 0.1 µg/mL ($VGCV_{0.1}$) dissolved in PBS (PBS) in apple juice (Appel J.), respectively. Each data point indicates the mean ± standard deviation of one assay in duplicate. (**b**) Evaluation of the area under curves of the CI_n. Three independent experiments were performed in duplicate (*$p < 0.05$, **$p < 0.01$, ***$p < 0.001$).
