# Supplementary material for: Oral Administration of Valganciclovir Reduces Clinical Signs, Virus Shedding and Cell-Associated Viremia in Ponies Experimentally Infected with the Equid Herpesvirus-1 C2254 Variant
Source: Pathogens. 2022 May 4;11(5):539. doi: 10.3390/pathogens11050539 (PMC9148010; doi:10.3390/pathogens11050539)

CONSORT

TRANSPARENT REPORTING of TRIALS

CONSORT 2010 Flow Diagram: Oral administration of valganciclovir reduces clinical signs, virus shedding and cell-associated viremia in ponies experimentally infected with the equid herpesvirus-1 C_{2254} variant.

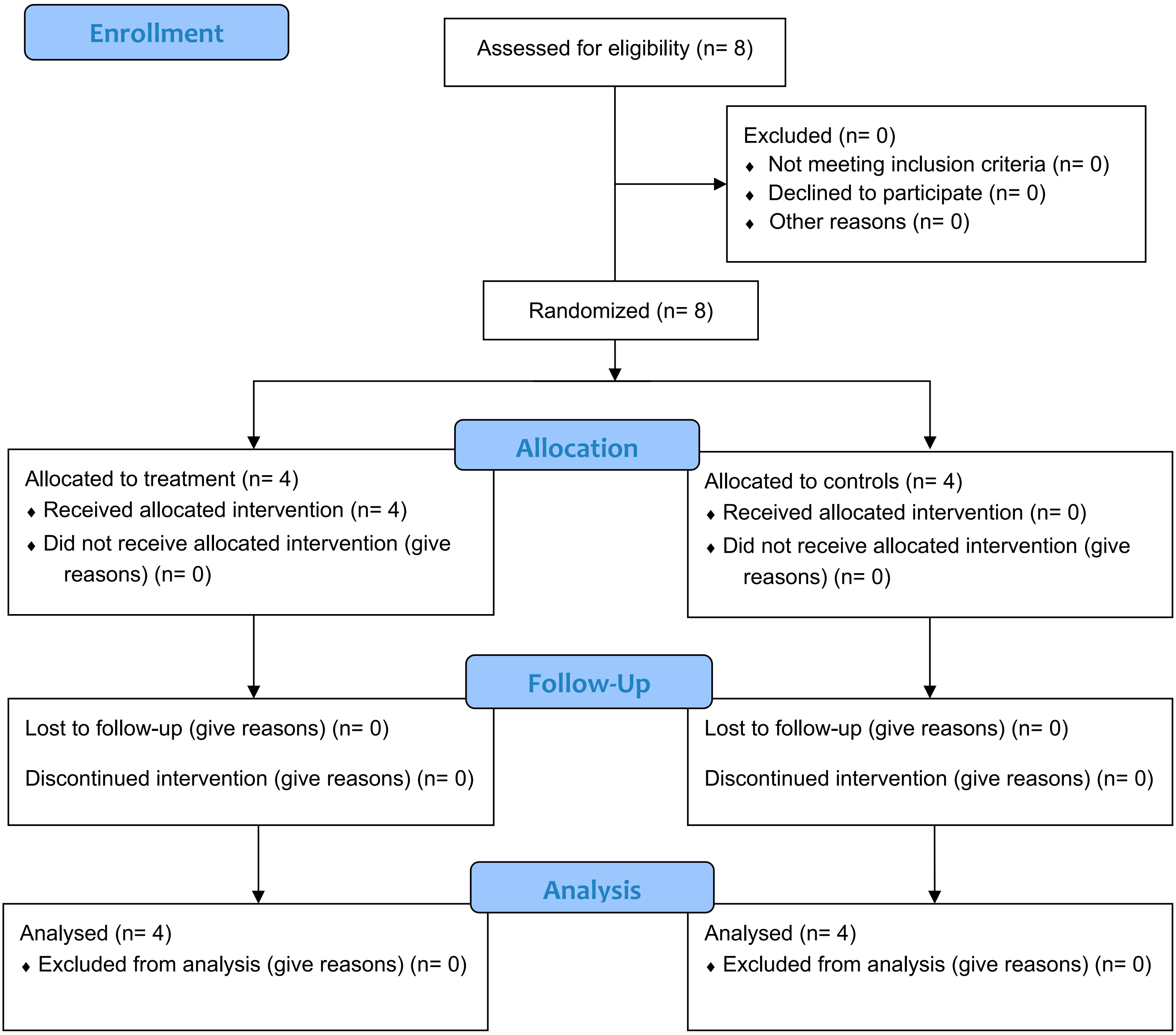

Supplement: Supplementary file 1 [file pathogens-11-00539-s001.zip › Figure S3.pdf]
