# Supplementary material for: Oral Administration of Valganciclovir Reduces Clinical Signs, Virus Shedding and Cell-Associated Viremia in Ponies Experimentally Infected with the Equid Herpesvirus-1 C2254 Variant
Source: Pathogens. 2022 May 4;11(5):539. doi: 10.3390/pathogens11050539 (PMC9148010; doi:10.3390/pathogens11050539)
Supplement: Supplementary file 1 [file pathogens-11-00539-s001.zip › Figure S4.pdf]

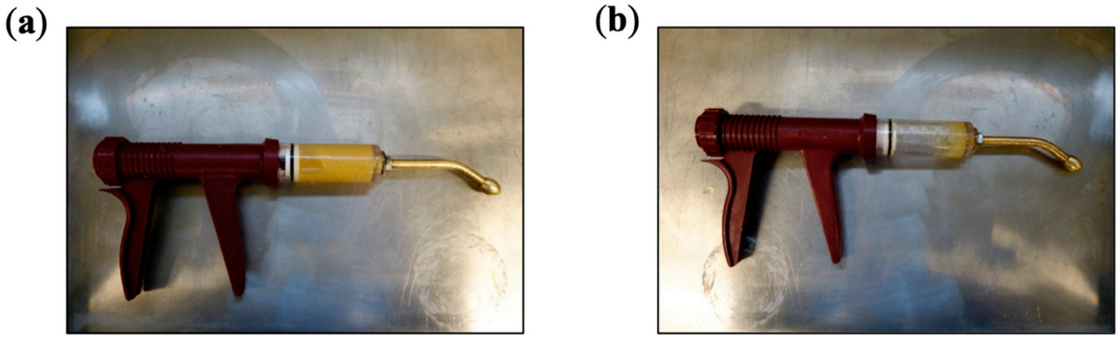

Figure S4. (**a**) Drug gun loaded with apple compote and apple juice ± valganciclovir. (**b**) Drug gun after treatment administration to ponies. A small volume of apple compote remains in the drug gun after each utilisation and was estimated to be less than 10% of initial volume. One specific drug gun was affected for each group for the duration of the study.
