# Supplementary material for: Oral Administration of Valganciclovir Reduces Clinical Signs, Virus Shedding and Cell-Associated Viremia in Ponies Experimentally Infected with the Equid Herpesvirus-1 C2254 Variant
Source: Pathogens. 2022 May 4;11(5):539. doi: 10.3390/pathogens11050539 (PMC9148010; doi:10.3390/pathogens11050539)
Supplement: Supplementary file 1 [file pathogens-11-00539-s001.zip › Table S1.pdf]

			**Nasopharyngeal swab**				**Blood**			
		**Pathogens/SNP**	**D+5**	**D+10**	**D+16**	**D+20**	**D+5**	**D+10**	**D+16**	**D+20**
**Treated group**	**Pony A**	EHV-1 A_{2254}	/	/	/	/	/	/	/	/
		EHV-1 G_{2254}	/	/	/	/	/	/	/	/
		EHV-1 C_{2254}	27.4	31.2	37.2	/	/	34.5	37.9	/
		EHV-4	/	/	/	/	/	/	/	/
	**Pony B**	EHV-1 A_{2254}	/	/	/	/	/	/	/	/
		EHV-1 G_{2254}	/	/	/	/	/	/	/	/
		EHV-1 C_{2254}	28.2	33.7	36.4	38.1	/	35.4	/	/
		EHV-4	/	/	/	/	/	/	/	/
	**Pony C**	EHV-1 A_{2254}	/	/	/	/	/	/	/	/
		EHV-1 G_{2254}	/	/	/	/	/	/	/	/
		EHV-1 C_{2254}	27.8	24.5	37.8	39.9	/	36.5	/	/
		EHV-4	/	/	/	/	/	/	/	/
	**Pony D**	EHV-1 A_{2254}	/	/	/	/	/	/	/	/
		EHV-1 G_{2254}	/	/	/	/	/	/	/	/
		EHV-1 C_{2254}	27.3	28.7	37.8	28.6	/	33.8	/	/
		EHV-4	/	/	/	/	/	/	/	/
**Control group**	**Pony E**	EHV-1 A_{2254}	/	/	/	/	/	/	/	/
		EHV-1 G_{2254}	/	/	/	/	/	/	/	/
		EHV-1 C_{2254}	24.5	28.5	37.3	39.1	/	32.1	/	/
		EHV-4	/	/	/	/	/	/	/	/
	**Pony F**	EHV-1 A_{2254}	/	/	/	/	/	/	/	/
		EHV-1 G_{2254}	/	/	/	/	/	/	/	/
		EHV-1 C_{2254}	24.8	30.5	33.8	38.4	/	31.5	36.4	/
		EHV-4	/	/	/	/	/	/	/	/
	**Pony G**	EHV-1 A_{2254}	/	/	/	/	/	/	/	/
		EHV-1 G_{2254}	/	/	/	/	/	/	/	/
		EHV-1 C_{2254}	24.0	31.3	36.1	/	/	33.0	38.9	/
		EHV-4	/	/	/	/	/	/	/	/
	**Pony H**	EHV-1 A_{2254}	/	/	/	/	/	/	/	/
		EHV-1 G_{2254}	/	/	/	/	/	/	/	/
		EHV-1 C_{2254}	25.0	33.3	36.6	39.6	/	30.8	37.3	/
		EHV-4	/	/	/	/	/	/	/	/

Each value corresponds to a Ct value determined by qPCR (Ct ≥ 40 were considered negative).
SNP: Single Nucleotide Polymorphism; A_{2254}: Arginine at position 2254 in the EHV-1 DNA polymerase; G_{2254}: Guanine at position 2254 in the EHV-1 DNA polymerase; C_{2254}: Cytosine at position 2254 in the EHV-1 DNA polymerase; “/”: no signal detected.
