# Supplementary material for: Oral Administration of Valganciclovir Reduces Clinical Signs, Virus Shedding and Cell-Associated Viremia in Ponies Experimentally Infected with the Equid Herpesvirus-1 C2254 Variant
Source: Pathogens. 2022 May 4;11(5):539. doi: 10.3390/pathogens11050539 (PMC9148010; doi:10.3390/pathogens11050539)
Supplement: Supplementary file 1 [file pathogens-11-00539-s001.zip › Table S2.pdf]

Clinical signs	Score	Description
Behaviour	0	Normal
	1	Quiet and nearly lethargic
	2	Severe depression
Rectal temperature	0	Normal (≤ 38.8 °C)
	1	Low fever (> 38.8 °C to ≤ 39.4 °C)
	2	Moderate fever (>39.4°C to ≤ 40.0 °C)
	3	Severe fever (> 40.0 °C)
Nasal discharge	0	No discharge
	1	Slightly and unilateral discharge
	2	Slightly and bilateral discharge
	3	Abundant and bilateral discharge or abundant and unilateral muco-purulent discharge
	4	Abundant and bilateral muco-purulent discharge
Cough	0	No coughing
	1	Coughing once during 30 min.
	2	Coughing two or more times during 30 min.
Ocular discharge	0	No discharge
	1	Slightly to moderate discharge
	2	Abundant discharge
Mandibular lymph node	0	Barely palpable
	1	Green peas-sized
	2	Bean-sized
	3	nuts sized
