# Supplementary material for: Oral Administration of Valganciclovir Reduces Clinical Signs, Virus Shedding and Cell-Associated Viremia in Ponies Experimentally Infected with the Equid Herpesvirus-1 C2254 Variant
Source: Pathogens. 2022 May 4;11(5):539. doi: 10.3390/pathogens11050539 (PMC9148010; doi:10.3390/pathogens11050539)
Supplement: Supplementary file 1 [file pathogens-11-00539-s001.zip › Table S3.pdf]

Virus	Target gene	Primers and probe sequences (5'-3')	Product size (bp)	Thermoprofile		Reference
EHV-1	Viral DNA polymerase (ORF30)	F: CCACCCTGGCGCTCG<br>R: AGCCAGTCGCGCAGCAAGATG<br>P (G_{2254}): FAM-TCC GTC GAC TAC TC-MGB-NFQ<br>P (A_{2254}): VIC-CATCCGTCAACTACTC-MGB-NFQ<br>P (C_{2254}): NED-CATCCGTCCACTACTC-MGB-NFQ	145	95 °C (10 min.)<br>95 °C (15 sec.)<br>62°C (1 min.)	X55	Allen *et al.*, 2007<br>&<br>Sutton *et al.*, 2020
EHV-1	gB	F: CATGTCAACGCACTCCCA<br>R: GGGTCGGGCGTTTCTGT<br>P: FAM-CCCTACGCTGCTCC-MGB-NFQ	63	95 °C (10 min.)<br>95 °C (15 sec.)<br>62°C (1 min.)	x45	Diallo *et al.*, 2006
EHV-4	gB	F: GGGCTATTGGATTACAGCGAGAT<br>R: TAGAATCGGAGGGCGTGAAG<br>P: VIC-CAGCGCCGTAACCAG-MGB-NFQ	58	95 °C (10 min.)<br>95 °C (15 sec.)<br>60°C (1 min.)	x45	Diallo *et al.*, 2007
EHV-2	gB	F: CCTCAACCTGACTGACATACCCA<br>R: TTTAGAATAGAGCTCAATCACCTTAAAATC<br>P: FAM-CACCATGATTACCCTGAACCTGTCTCTGGT-BHQ1	100	95 °C (10 min.)<br>95 °C (15 sec.)<br>60°C (1 min.)	x45	Brault *et al.*, 2010
EHV-5	gB	F: AACCCGCCGTGCATCA<br>R: AGGCGCCACACACCCTAA<br>P: FAM-ACAACACCACCAACCCCTTTCTGCTG-BHQ1	66	95 °C (10 min.)<br>95 °C (15 sec.)<br>60°C (1 min.)	x45	Hue *et al.*, 2014
EIV	M	F: AGATGAGYCTTCTAACCGAGGTCG<br>R : TGCAAANACATCYTCAAGTCTCTG<br>P: FAM-TCAGGCCCCCTCAAAGCCGA-BHQ1	102	95 °C (10 min.)<br>95 °C (15 sec.)<br>60°C (1 min.)	x45	Heine *et al.*, 2007

bp: base pair; EHV: Equid Herpesvirus; EIV: Equine Influenza Virus; gB: glycoprotein B; ORF: Open Reading Frame; F: Forward; R: Reverse; P: Probe
